# Predicting Initial Client Engagement with Community Mental Health Services by Routinely Measured Data

**DOI:** 10.1007/s10597-014-9740-9

**Published:** 2014-07-02

**Authors:** Diana Roeg, Ien van de Goor, Henk Garretsen

**Affiliations:** Department Tranzo, Tilburg University, PO Box 90153, 5000 LE Tilburg, The Netherlands

**Keywords:** Assertive outreach, Interferential care, Community mental health, Engagement

## Abstract

Engagement is a determinant of how well a person will respond to professional input. This study investigates whether, in practice, routinely measured data predict initial client engagement with community mental health services. Engagement, problem severity, client characteristics, and duration before the first contact were measured at team entrance with clients (n = 529) of three community mental health teams. Regression analysis was used to predict engagement. Gender, age, referrer, having children, having a partner, and ethnicity showed a minor relationship with engagement. Higher problem severity measured by the team members with the Health of the Nation Outcome Scales, being referred for having psychiatric problems and/or causing severe and long-lasting trouble (as ‘assessed’ by the often non-professional referrer), and a longer duration between enrollment and the first conversation with a client, were indicative for a lower engagement. The final model explained 19.2 % of the variance in engagement. It can be concluded that initial client engagement with community mental health services can be predicted, in part, by routinely measured data. The findings can be used by community mental healthcare teams to create an awareness system.

## Introduction

Community mental health services are in place to support persons with severe psychiatric and/or addiction problems who are hard to engage with healthcare services whilst living in the community (Rapp 1998; Test and Stein 2000; Thompson et al. 1990; Wingerson and Ries 1999). In community mental health services patients are treated where they live. Assertive outreach, an important part of these services, refers to the active ‘seeking out’ and engagement with clients (Burns 2002; Wingerson and Ries 1999).

Engagement is a determinant of how well a person will respond to professional input (Toynbee and Allen 2008). Problems with engagement can lead to low involvement in care and to increased dropout, so that individuals with severe, long-term mental health problems may not receive adequate care in the community (Hall et al. 2001; Rush et al. 1999).

Therapeutic alliance is often mentioned as the quintessential element of engagement through which active collaboration and participation occurs. However, besides the (suboptimal) relationship with the service provider (e.g. not feeling listened to), clients themselves often mention their mental illness (resulting in a loss of autonomy and identity) as the main reason for non-engagement (Priebe et al. 2005). Furthermore, client demographics and characteristics are reported to have a relationship with engagement (Bradley 2006).

The processes of engagement/disengagement have gained increasing interest in community mental health services (Gillespie et al. 2004; Meaden et al. 2004; Paget et al. 2009; Priebe et al. 2005; Tait et al. 2003; Toynbee and Allen 2008). Most studies focused on the relation between engagement and outcomes, and very few on the prediction of engagement. Currently, one difficulty is that engagement is conceptualized and measured in different ways; this confuses the validity of the findings and makes it difficult to compare the results of studies on this topic. In a literature review, Bradley (2006) showed that there is no clear working definition of the concept of engagement; instead, engagement is interpreted from collaboration to (medication) compliance. According to Bradley (2006) engagement is a multi-dimensional concept including not only interest formation or compliance to predefined plans, but also the development of trust, rapport and ongoing involvement. Engagement comprises the process where clients are actively involved in collaboration and participation. In the absence of a widely accepted definition, several standardized measures have been developed to determine the level of engagement (e.g., Gillespie et al. 2004; Hall et al. 2001; Tait et al. 2002). The first validated tool was the Engagement Measure (Hall et al. 2001), assessing engagement from a staff perspective and including six dimensions of engagement: appointment keeping, client-therapist interaction, communication/openness, client’s perceived usefulness of treatment, collaboration with treatment, and compliance with medication. The Engagement Measure was based on an existing monitoring form of an assertive outreach practice and on discussions with 13 healthcare professionals. Subsequently, based on the Engagement Measure, a tool from the client’s perspective was developed by Gillespie et al. (2004). The Service Engagement Scale is a staff-rated instrument including four dimensions: availability, collaboration, help seeking and treatment adherence, and was developed based on literature research and discussions with two healthcare professionals (Tait et al. 2002).

For community mental healthcare team members, it would be very supportive if they could predict and improve the engagement of their clients in an early stage. Based on the above-mentioned evidence, the first recommendation for them would be to invest in the relationship with their clients. Second, it would be helpful if they had knowledge about the other factors, detectable in an early stage of the therapeutic relationship, that influence the initial engagement of clients. Such increased insight can help to predict which clients will benefit most from the services offered by community mental health teams, and can enhance individual treatment planning. Moreover, such insight can help in developing interventions to support community mental healthcare team members to improve engagement with the clients for whom this is difficult to realize.

The present study focuses on the prediction of initial engagement of clients with community mental health services. Because little is known about the background of clients in the early stages of bonding, this study includes information to predict engagement that is available for staff members at that point in time, i.e. routinely measured data. For the assessment of engagement in that early period, a valid and easy to use tool is required that reflects all relevant aspects of the concept. As the staff-rated Engagement Measure (Hall et al. 2001) was based on extensive data collection, reflects six dimensions of engagement and is relatively short, this measure is considered to be the most useful instrument for the purpose of the present study.

## Methods

### The Teams

This study includes three community healthcare teams from different regions that target marginalized persons with problems in multiple life areas who are not yet involved in any healthcare trajectory. These so-called interferential care teams are interdisciplinary and include staff from several organizations (i.e., mental healthcare, addiction care, welfare work, general healthcare services, centers for the homeless, and care for the mentally disabled). Staff members have shared responsibility, meaning they often visit clients in couples and discuss the caseload in regular team meetings. Clients do not enroll themselves but are introduced to the teams by e.g. family members, healthcare institutions, housing corporations, and others (i.e. the referrers). After a referral, the staff members collect information about the client in their surroundings and during home visits. The major aim of the interferential care teams is to make and retain contact with clients and establish a bond, and (after a number of months) prepare them for and link them to existing regular healthcare services. The services are outreaching and comprise practical support. Although the teams do not provide treatment, a psychiatrist can be consulted by the team members. Interferential care teams are common in the Netherlands and have existed for over 25 years; recently, 277 teams were identified (Roeg et al. 2007). The main aim of interferential care teams is to engage persons in healthcare services that are currently out of reach. The reasons for non-engagement remain unclear: some suggest an inability or unwillingness of clients, whereas others suggest an inability of the healthcare system to commit some clients (Schout et al. 2011).

### Participants

All clients that entered the three interferential care teams between November 2008 and April 2011 were included in the study. The Engagement Measure and the predictors were all routinely measured by the team as part of daily practice. The research team received an encrypted dataset including anonymous data of the clients.

### Measures

Assessments were made within the first few weeks after the first contact with the client, i.e. as soon as the involved service providers had sufficient information about the client to answer the items of the observer-rated measures. The best informed staff members, often two, filled out the assessments.

The following measures were included:

#### Outcome

Engagement was assessed with the 11-item Engagement Measure (Hall et al. 2001). This requires that the most involved staff member rates a client on six dimensions using a 5-point response scale, ranging from 1 (‘Always’, e.g., always keeps appointments) to 5 (‘Never’, e.g., never keeps appointments). According to Hall et al. (2001), an overall engagement score of ≥33 indicates progressively good engagement whilst scores <33 indicate relatively poor engagement. The Engagement Measure has demonstrated good internal consistency (α = 0.89), inter-rater reliability (α = 0.95) and test–retest reliability (α = 0.90) (Hall et al. 2001). For the present study, the original Engagement Measure was translated into Dutch and some items were adjusted to match actual/local practice; all adjustments were minor and involved phrasing of words only.

#### Predictors

First, items on client characteristics filled in by the staff covered client demographics: gender, age (based on date of birth), having children (y/n), having a partner (y/n), and ethnicity (Dutch or other). Second, the problem areas which caused the referrer to contact the interferential care team are registered. Other registered items that were hypothesized to be related to initial engagement included: third, the referrer, and fourth, the duration (the number of weeks and number of attempts) required to achieve a first conversation with a client.

Fifth, problem severity was measured by the staff using the Health of the Nation Outcome Scales (HoNOS); this is a 12-item instrument including dimensions on behavioral problems (including overactive and aggressive behavior, self-injury, problem drinking or drug-taking), impairment (including cognitive problems, physical illness or disability), symptomatic problems (including three types of psychiatric symptoms) and social problems (including problems with relationships, activities of daily living, living conditions, and occupation and activities). Items are scored on a 5-point response scale, ranging from 0 (no problem) to 4 (severe to very severe problem) and sum scores are used to reflect the total severity of problems (Mulder et al. 2004; Wing et al. 1998). A lower score indicates lower problem severity. The internal consistency of the HoNOS is moderately high (Cronbach’s α 0.59–0.76) (Pirkis et al. 2005). To enlarge inter-rater reliability, as is recommended, staff in the present study received a 4-h training from an official HoNOS instructor (Brooks 2000). The difference compared with the variable ‘problem area according to the referrer’ is that the HoNOS provides a systematic assessment and is based on a professional view.

### Analysis

Standard multiple regression was used. Preliminary analyses were conducted to ensure there was no violation of the assumptions of normality, linearity, multicollinearity and homoscedasticity.

Because data collection was part of the routine outcome monitoring and clients received the services they would normally receive, according to the central committee on human research (CCMO), no medical ethical approval was required for this study. At discharge, clients were notified by their service provider about this study and they were given the opportunity to refuse to allow their data to be used for this purpose by returning a reply card.

There are no known conflicts of interest. All authors certify responsibility for this article.

## Results

### Participant Characteristics

The dataset included 529 clients. One client refused inclusion of his data in the study, two clients in the dataset appeared to be (mistakenly) double entered, and for three clients no data were entered. Therefore, the final data set included 523 clients. The mean age of the clients is 46 years, 66 % are male and most are single. Most clients are referred to the teams by family/friends, housing corporations or municipalities. The most common problem areas mentioned by the (often non-professional) referrers involve financial issues, psychiatric disorders, and/or addiction. On average it takes about 2 weeks to get in contact with these clients. Mean score on the Engagement Measure is 40.65 indicating progressively good engagement, and 14.58 on the HoNOS indicating a problem severity comparable to that of psychiatric clients in day care (Table 1).

**Table 1:** Demographic and service characteristics of the study clients

	Total n = 523
Age in years: mean (SD)	46 (16)
Male (%)	66
Non-Dutch (%)	14
With children (%)	38
With a partner (%)	18
Engagement Measure score: mean (SD)	40.65 (9.41)
HoNOS score: mean (SD)	14.58 (6.63)
Referrer(s) (%)	
Family/friends	18
Housing corporation	16
Municipality	14
Police	11
Primary physician	8
Social work	8
Addiction care	5
Mental health	5
Form of cooperation between local or regional social organisations	3
Other community mental health team	3
Community shelters	3
Neighbours	3
School/work	2
Child welfare	2
Care for mentally disabled	2
Area health authority	1
Other	11
No. of problem area(s) according to referrer: mean (SD)	3.1 (1.4)
Problem area(s) according to referrer (%)	
Financial	49
Psychiatric	44
Addiction	43
Filthy and neglected	37
Social contacts	33
Day-time activities	27
Causing severe and long lasting trouble	22
Somatic	17
Homeless	16
Mentally disabled	10
Criminal activities	6
Other	4
No. of weeks before first conversation with a client: means (SD)	2.29 (3.46)
No. of attempts to get to a first conversation with a client: mean (SD)	1.96 (2.4)

Individual HoNOS items (Table 2) show that most clients have problems that need action (a score of ≥2) on the subscale ‘social problems’, including the items on relationships, activities of daily living, living conditions, and occupation and activities. Additionally, many clients score ≥2 on problem drinking or drug taking. Furthermore, 25 % of the clients have severe to very severe problems (a score of 4) with living conditions (homeless or at high risk to become homeless). Lower scores are seen for non-accidental self-injury, cognitive problems, problems associated with hallucinations/delusions, and problems with depressed mood.

**Table 2:** Data on problem severity measured with the HoNOS

HoNOS items	Mean (SD)	Percentage scoring 2 and over^a^	Percentage scoring 4^b^
1. Overactive, aggressive, disruptive or agitated behavior	0.80 (1.24)	23.7	5.8
2. Non-accidental self-injury	0.15 (0.59)	4.7	0.8
3. Problem drinking or drug taking	1.63 (1.52)	50.0	15.4
4. Cognitive problems	0.93 (1.26)	29.1	5.4
5. Physical illness or disability problems	1.21 (1.39)	36.7	8.3
6. Problems associated with hallucinations and delusions	0.43 (1.09)	12.2	5.4
7. Problems with depressed mood	1.24 (1.23)	43.0	4.4
8. Other mental and behavioral problems	1.05 (1.47)	33.3	10.2
9. Problems with relationships	2.05 (1.34)	67.4	17.6
10. Problems with activities of daily living	1.63 (1.37)	53.1	10.8
11. Problems with living conditions	1.85 (1.66)	54.0	25.1
12. Problems with occupation and activities	1.60 (1.47)	49.4	13.9

^a^A score of 2 and over indicates there is a problem needing action

^b^A score of 4 indicates severe to very severe problems

### Prediction of Engagement

After the preliminary analyses, the predictors: (1) demographics including gender, age, having children, having a partner and ethnicity, and (2) referrer were excluded from the model because they showed only a minor relationship (β ≤ 0.1) with the dependent variable. The final model, which includes: (1) client’s problem areas according to referrer, (2) problem severity measured with the HoNOS, and (3) both the number of weeks and number of attempts to get to a first conversation with the client, explains 19.2 % of the variance in engagement (*p* < 0.001). Of these variables, problem severity (as measured by professionals with the HoNOS) (β = −0.271, *p* < 0.001) and number of weeks to get to a first conversation with a client (β = −0.143, *p* = 0.004) make the largest unique significant contribution, followed by the problem areas according to referrer: ‘psychiatric’ (β = −0.110, *p* = 0.017) and ‘causing severe and long lasting trouble’ (β = −0.114, *p* = 0.007) (Table 3).

**Table 3:** Final model of the multiple regression analysis: the contribution of routinely measured variables in the prediction of engagement

	B	Standard error	β	*p* value
(Constant)	48.593	1.164		<0.001*
Problem areas according to referrer				
Psychiatric	−2.089	0.875	−0.110	0.017*
Financial	0.535	0.831	0.028	0.520
Somatic	2.451	1.079	0.099	0.024*
Mentally disabled	2.986	1.340	0.096	0.026*
Addiction	−0.503	0.840	−0.027	0.549
Homeless	−1.474	1.142	−0.057	0.197
Filthy and neglected	−0.767	0.875	−0.039	0.381
Day time activities	1.107	0.933	0.052	0.236
Social contacts	−2.56	0.876	−0.013	0.770
Criminal activities	0.629	1.722	−0.016	0.715
Causing severe and long lasting trouble	−2.591	0.956	−0.114	0.007*
Other	4.126	2.115	0.082	0.052
No. of weeks to get to a first conversation with a client	−0.388	0.132	−0.143	0.004*
No. of attempts to get to a first conversation with a client	−0.327	0.189	−0.085	0.084
Problem severity (HoNOS)	−3.84	0.66	−0.271	<0.001*

* Significant at *p* < 0.05. R^2^ = 0.192

## Discussion

This study aimed to elucidate the relationship between routinely measured variables and engagement of clients receiving care from interferential care teams (i.e. community mental health teams).

### Client Characteristics

The clients in this study had a mean initial engagement score of 40.65, which is relatively high considering that an overall engagement score of ≥33 indicates progressively good engagement (Hall et al. 2001). In contrast, the mean HoNOS score (assessing problem severity on multiple areas from a professional point of view) of 14.58 indicates that these clients have rather severe problems, comparable to the level of problems of psychiatric clients receiving day care (Mulder et al. 2004). The high level of problems is a serious concern, considering that the clients in the present study have only recently been retrieved by the interferential care teams and have not previously received any kind of healthcare service. It is noteworthy that, despite the high level of problem severity, these clients still show relatively high levels of initial engagement, showing that, once in contact, clients were apparently willing to cooperate with the team members. The findings therefore suggest that these clients are not as unwilling to receive services as previously thought, but that there are other reasons why they are not making use of the available facilities (Roeg et al. 2013). For example, it might be too difficult for them to start making use of or comply with the regular services provided (e.g. due to waiting lists, administration, keeping appointments, method of approach of service providers and office-based provision of services), or services/staff members are not adjusted to the problems of these clients (multiple in nature) or their way of behaving, as was suggested by Schout et al. (2011). The present results indicate that the approach of the interferential care team members seems to be the right answer in resolving the engagement problem within this group of clients.

### Predictors of Engagement

The demographics, including gender, age, having children, having a partner, ethnicity, and referrer showed minor correlation with the dependent variable and were deleted from the model. It is concluded that these predictors do not play a role in explaining differences between clients in initial engagement. One might expect that language or cultural differences can be experienced as a barrier in engaging clients with a non-Dutch nationality. However, for gender, having a partner, and ethnicity our findings are consistent with other studies showing no relationship between these predictors and engagement (Bradley 2006; Hall et al. 2001; Klinkenberg et al. 1998; Mowbray et al. 1993; Mulder et al. 2005; Tait et al. 2003). It is possible that the low threshold approach, or the practical nature of the services in interferential care, are helpful factors in overcoming ethnic differences and gaining trust. However, in contrast to our study, in some studies age and having children are reported to have a positive relationship with engagement (Bradley 2006; Draine and Soloman 1996; Fiorentine et al. 1999; Greeno et al. 1999; Hall et al. 2001; Klinkenberg et al. 1998; Sainsbury Centre for Mental Health 1998; Tait et al. 2003). The differences in these outcomes might be attributed to the fact that engagement was measured in different ways in the various studies.

The final model explained 19.2 % of the variance in the engagement scores. Problem severity measured with the HoNOS, and the number of weeks required to get to a first conversation with a client, made the largest unique significant contribution, followed by the problem areas, ‘causing severe and long-lasting trouble’ and ‘psychiatric problems’ mentioned by the referrers. All these variables have a negative relation with the total engagement scores; this implies that more severe problems measured with the HoNOS, a longer duration before the first contact, being referred to the teams for having psychiatric problems, and/or causing severe and long-lasting trouble (as assessed by often non-professional referrers) all lead to a lower engagement. These findings confirm qualitative findings in which mental illness (and subsequent loss of autonomy and identity as a part of the experience of mental illness) were mentioned by clients as a main factor for suboptimal engagement (Priebe et al. 2005). Our findings are also in line with a study in which lower Global Assessment of Functioning scores were found to be negatively associated with engagement (Bradley 2006). In addition, problems with the criminal justice system and substance abuse are also reported to have a negative relation with engagement (Greeno et al. 1999; Klinkenberg et al. 2002; Mulder et al. 2005); however, these latter findings were not replicated in our study. An explanation for the negative relation between number of weeks to get to a first conversation with a client and engagement might be that a longer duration before the first contact indicates difficulties in engaging the person and could be a sign that more than ‘regular’ engagement activities are needed. In the present study, although the predictors included in the model explain part of the variation in engagement scores, 80.8 % of the variation still remains unexplained. For this variation an explanation needs to be sought in variables other than those examined in this study.

Community mental healthcare teams can benefit from the findings of this study. Team members can use this information to create an awareness system that helps them to recognize which clients might be more difficult to engage than others. The team can try to anticipate the problems and adapt their engagement activities in an early stage. When clients have a higher HoNOS score and/or are referred due to causing severe and long-lasting trouble and/or have psychiatric problems, this is an early indication that engagement might need more attention. Furthermore, when weeks pass and no first conversation has taken place with a client, this is also a sign that engagement needs extra consideration. When there are indications for a lower expected engagement, the team might discuss the engagement of this client in a team meeting to decide on the actions required. The client may need increased attention, another approach, or a change of team member. Including the items of the Engagement Measure in this discussion can be useful.

It can be concluded that initial client engagement with community mental healthcare services can, to some extent, be predicted. Higher problem severity measured with the HoNOS and being referred for having psychiatric problems and/or causing severe and long-lasting trouble (as indicated by the often non-professional referrers) are indicative for a lower engagement. Also, a negative relation was found between ‘number of weeks to get to a first contact with a client’ and engagement. Community mental healthcare teams can use these findings to create an awareness system.

### Study Limitations

This study has some limitations that need to be addressed. First, the translation of the Engagement Measure into Dutch was forward only and some minor modifications were made to the original UK version to realize a better fit with the Dutch interferential care practice. The first author performed the translation based on conversations with the teams about the terminology they used; the other two authors checked the translation and meaning by comparing it with the original instrument. Modifications included terminology: ‘key worker’ (overall) became *bemoeizorger* (meaning: interferential care provider), ‘therapist’ (item 3) became ‘*bemoeizorger*’, and ‘treatment and homework’ (item 5) became ‘*begeleiding en voorgestelde acties en behandeling’* (meaning: care provision and advised actions and treatment). Layout was adapted: items were put in a table to reduce repetition in phrasing in the response scales, and coding was inversed according to the remainder of the instruments used (and recoded again in the analyses). Therefore, compared with the outcomes measured with the UK version, there may be some minor differences in interpretation.

In addition, the findings of the present study apply to a specific group of clients investigated within a specific social context; this implies that the present findings may not be generalized to other clients and contexts. This means that the findings only apply to clients who have severe problems in multiple life areas and are difficult to reach by regular healthcare services and, for that reason, are approached by community-based services. As interferential care teams provide care that is comparable to community mental healthcare teams in other countries, the findings are most likely generalizable to the clients of these teams. Additionally, when interpreting the findings, we should be aware of some form of selection effect. Selection bias due to the research design is not likely as no pre-selection of clients took place but, instead, all clients of the teams were initially included in the study. However, we should keep in mind that the HoNOS and Engagement Measure (both ROM measures) were not filled out for all clients. The team registrations did not allow us to calculate exact completion rates^1^. Instead, we asked the data coordinators of the teams to make a rational estimation. They were asked to take a random sample of the clients and look for indications that were available in the registrations (e.g. time between registration and deregistration, type of activities undertaken). In the three teams, the average completion rate was estimated to be 54 %. In comparison, regular ROM response rates in mental healthcare are reported to be around 35 % (Van Ham and Reitsma 2011). In the teams, the reasons for not filling out ROM measures included: still unfamiliar with the use of measurement instruments in daily practice [ROM in interferential care teams is relatively new], not enough information about the client, no time, and client is difficult to approach.

Finally, as with all cross-sectional studies, no causality can be proven. In the model tested, problem severity measured with the HoNOS explains a significant part of the variation in initial engagement. However, despite a clear relationship between problem severity and engagement, because they were measured at the same moment in time we cannot be absolutely certain which variable occurred earlier in time than the other. Although not plausible, theoretically it could be that engagement predicts problem severity. More studies are needed to further elucidate these factors. In either case, however, the advice to the team members would remain the same because, without engagement, a care provider cannot make a difference in someone’s life. Furthermore, using the HoNOS score as input for an awareness system can never be wrong, as extra attention paid to the engagement of a person is not expected to have negative consequences as long as it is carried out with respect for the person involved. Therefore, the above-mentioned advice of being aware that clients with a high HoNOS score (indicating high problem severity) might need more attention to engage, still holds.

## Footnotes

1 This is because only a proportion of all clients that are registered indeed received an interferential care trajectory and were to be included in the study. The other part includes persons that appear not to be in need of interferential care; for instance, after some investigation it appears that they do not have sufficiently severe problems to warrant interferential care. Other clients appear to be under treatment elsewhere. Data regarding which clients indeed received an interferential care trajectory cannot be retrieved from the registrations, because these included all clients that are initially reported to the teams and no markers are included to help distinguish specific clients with an interferential care trajectory.

## References

[CR1] Bradley S (2006). Engagement in assertive outreach: Compliance or alliance?.

[CR2] Brooks R (2000). The reliability and validity of the Health of the Nation Outcome Scales: Validation in relation to patient derived measures. Australian and New Zealand Journal of Psychiatry.

[CR3] Burns T (2002). The UK700 trial of intensive case management: An overview and discussion.

[CR4] Draine J, Soloman P (1996). Case manager alliance with clients in an older cohort. Community Mental Health Journal.

[CR5] Fiorentine R, Nakashima J, Anglin M (1999). Client engagement in drug treatment. Journal of Substance Abuse Treatment.

[CR6] Gillespie M, Smith J, Meaden A, Jones C, Wane J (2004). Clients’ engagement with assertive outreach services: A comparison of client and staff perceptions of engagement and its impact on later engagement. Journal of Mental Health.

[CR7] Greeno CG, Anderson CM, Shear MK, Mike G (1999). Initial treatment engagement in a rural community mental health centre. Psychiatric Services.

[CR8] Hall M, Meaden A, Smith J, Jones C (2001). Brief report: The development and psychometric properties of an observer-rated measure of engagement with mental health services. Journal of Mental Health.

[CR9] Klinkenberg W, Caslyn R, Morse G (1998). The helping alliance in case management for homeless persons with severe mental illness. Community Mental Health Journal.

[CR10] Klinkenberg W, Caslyn R, Morse G (2002). The case managers view of the helping alliance. Care Management Journals.

[CR11] Meaden A, Nithsdale V, Rose C, Smith J, Jones C (2004). Is engagement associated with outcome in assertive outreach?. Journal of Mental Health.

[CR12] Mowbray CT, Cohen E, Bybee D (1993). The challenge of outcome evaluation in homeless services: Engagement as an intermediate outcome measure. Evaluation and Program Planning.

[CR13] Mulder C, Koopmans G, Hengeveld A (2005). Lack of motivation for treatment in emergency psychiatry patients. Social Psychiatry and Psychiatric Epidemiology.

[CR14] Mulder CL, Staring ABP, Loos J, Buwalda VJA, Kuijpers D, Sytema S (2004). De Health of the Nation Outcome Scales (HoNOS) als instrument voor ‘routine outcome assessment’. Tijdschrift voor Psychiatrie.

[CR15] Paget A, Meaden A, Amphlett C (2009). Can engagement predict outcome in assertive outreach. Journal of Mental Health.

[CR16] Pirkis JE, Burgess PM, Kirk PK, Dodson S, Coombs TJ, Williamson MK (2005). A review of the psychometric properties of the Health of the Nation Outcome Scales (HoNOS) family of measures. Health and Quality of Life Outcomes.

[CR17] Priebe S, Watts J, Chase M, Matanov A (2005). Processes of disengagement and engagement in assertive outreach patients: Qualitative study. British Journal of Psychiatry.

[CR18] Rapp CA (1998). The active ingredients of effective case management: A research synthesis. Community Mental Health Journal.

[CR19] Roeg DPK, van de Goor LAM, Garretsen HFL (2007). European approach to assertive outreach for substance users: Assessment of program components. Substance Use and Misuse.

[CR20] Roeg, D. P. K., van de Goor, L. A. M., Voogt, M. C. M., van Assen, M. A. L. M., & Garretsen, H. F. L. (2013). Effects of interferential care: A community-based care program for persons with severe problems on several life areas.* International Journal of Social Psychiatry*. doi:10.1177/0020764013507247. 10.1177/0020764013507247 PMC4230954 24221098

[CR21] Rush, B., Norman, R., Kirsh, B., & Wild, C. (1999). Explaining outcomes: Developing instruments to assess the critical characteristics of community support programs for people with severe mental illness. London [etc.]: Centre for Addiction and Mental Health, London Health Sciences Centre, University of Toronto, University of Alberta.

[CR22] Sainsbury Centre for Mental Health (1998). Keys to engagement: Review of care for people with severe mental illness who are hard to engage with services.

[CR23] Schout G, de Jong G, Zeelen J (2011). Beyond care avoidance and care paralysis: Theorizing public mental health care. Sociology.

[CR24] Tait L, Birchwood M, Trower P (2002). A new scale (SES) to measure engagement with community mental health services. Journal of Mental Health.

[CR25] Tait L, Birchwood M, Trower P (2003). Predicting engagement with services for psychosis: Insight, symptoms and recovery style. British Journal of Psychiatry.

[CR26] Test MA, Stein LI (2000). Practical guidelines for the community treatment of markedly impaired patients. Community Mental Health Journal.

[CR27] Thompson KS, Griffith EE, Leaf PJ (1990). A historical review of the Madison model of community care. Hospital & Community Psychiatry.

[CR28] Toynbee L, Allen D (2008). Non-engagement and the assertive outreach team. Psychiatric Bulletin.

[CR29] Van Ham, M., & Reitsma, E. (2011). Context—taal en vorm. (Context—language and form). In S. van Hees, P. van der Vlist & N. Mulder (Eds.), *Van weten naar meten. ROM in de GGz (From assessment to knowledge. ROM in mental healthcare)* (pp. 27–33). Amsterdam: Boom.

[CR30] Wing JK, Beevor AS, Curtis RH, Park SBG, Hadden S, Burns A (1998). Health of the Nation Outcome Scales (HoNOS); research and development. British Journal of Psychiatry.

[CR31] Wingerson D, Ries RK (1999). Assertive community treatment for patients with chronic and severe mental illness who abuse drugs. Journal of Psychoactive Drugs.

